# Current Trends and Future Directions for Outpatient Total Joint Arthroplasty: A Review of the Anesthesia Choices and Analgesic Options

**DOI:** 10.5435/JAAOSGlobal-D-22-00259

**Published:** 2023-09-06

**Authors:** Brian M. Osman, Tuan G. Tieu, Yosira Guevara Caceres, Victor H. Hernandez

**Affiliations:** From the Perioperative Medicine and Pain Management, University of Miami Health System, University of Miami Miller School of Medicine, Miami, FL (Dr. Osman, Dr. Caceres, and Dr. Hernandez); Jackson Memorial Hospital, University of Miami Miller School of Medicine, Miami, FL (Dr. Tieu).

## Abstract

The aging population and the obesity epidemic have led to an increased rate of joint arthroplasty procedures, specifically total knee arthroplasty and total hip arthroplasty. These surgeries are associated with increased hospital length of stay and, consequently, higher costs. Despite the benefits of outpatient surgery, only a small percentage of total joint arthroplasties (TJAs) are done in this manner. We reviewed the most up-to-date trends for outpatient TJA and discussed essential factors for a successful outpatient program, including the proper patient selection process and best available anesthetic and analgesic options, along with their risks and benefits. Risk stratification tools, such as the Outpatient Arthroplasty Risk Assessment, are helpful for predicting outcomes regarding outpatient TJA, and neuraxial anesthesia should be considered to minimize complications and facilitate early discharge. A multimodal analgesia regimen could be effective for pain management in outpatient TJA, and the currently recommended peripheral nerve blocks for total hip arthroplasty and total knee arthroplasty are the fascia iliaca compartment block and adductor canal block, respectively. However, blocks should be carefully considered for outpatient procedures. Enhanced recovery after surgery (ERAS) protocols help to guide perioperative care teams and allow for improved patient recovery, decreased length of stay, and increased patient satisfaction.

Total joint arthroplasty (TJA) is one of the most common surgeries done in the United States, and of these surgeries, the two most common ones are total knee arthroplasty (TKA) and total hip arthroplasty (THA). As a result of an aging population and the obesity epidemic leading to an increased incidence of osteoarthritis, estimates predict that primary TKA and THA surgeries will increase by 401% and 284%, respectively, by 2040. This equates to an estimated 3.4 million TKAs annually and 1.4 million THAs annually.^1^

TJA was cited in 2015 as one of the most expensive surgeries covered by Medicare, costing over $33,000 per surgery, equating to a payout of over $6.5 billion by Medicare in 2013.^2^ Increased hospital length of stay (LOS) also directly increases the total cost for TJA.^3^ Overall, continued advances in surgical, anesthetic, and analgesic techniques as well as evolving pathways and perioperative care teams focused on rapid postoperative recovery and rehabilitation have improved patient satisfaction and shortened postsurgical recovery times. However, in 2018, only 0.6 to 1% of primary TJAs were done on an outpatient basis and only 6.2 to 16.5% were discharged within 24 hours if admitted postoperatively.^2^

The recent interest in performing TJA as an outpatient procedure or with 23-hour observation has been fueled by the removal of TKA and TJA from the Inpatient Only (IPO) List by the Centers for Medicare & Medicaid Services (CMS), and also by the effects of the COVID-19 pandemic. This is feasible with proper patient selection, specific anesthetic and analgesic techniques, and a well-prepared perioperative team.^2^ Furthermore, an outpatient and ambulatory surgery center setting can reduce TJA costs by thousands of dollars per procedure.^4^

We will discuss the patient selection process to determine the best candidates for outpatient TJA. We will also review the available anesthetic and analgesic options, their outcomes, and efficacy and how they can be used for outpatient TJA.

## Patient Selection

One of the most important factors for a successful outpatient TJA program is proper patient selection, but the literature is mixed regarding consensus or guidelines. For example, Sher et al^5^ (2017) found that discharged patients within 24 hours of primary TJA were likely to be younger (age < 50 years), to be male, to be healthier [American Society of Anesthesiologists (ASA) score of 1 to 2], not morbidly obese (body mass index (BMI) < 40), and not on chronic steroid therapy and less likely to have chronic disease (cardiac, hypertension, diabetes, stroke, or coagulation disorders).

Conversely, various studies have looked at patient risk factors associated with increased rates of complications and hospital readmissions. A retrospective study by Courtney et al^6^ (2015) noted that most postoperative complications occurred >24 hours after the initial surgery with the following patient characteristics: advanced age (older than 60 years) and history of chronic obstructive pulmonary disease, congestive heart failure, or coronary artery disease.

Various scores and indices attempt to risk-stratify patients undergoing TJA and predict possible postoperative complications. For example, the Charlson Comorbidity Index and the ASA Physical Status Score consider the patient's cumulative comorbidities and disease burden. However, they have poor sensitivity and specificity in predicting outcomes regarding outpatient TJA. In response to this, the Outpatient Arthroplasty Risk Assessment (OARA) score was developed to provide a comprehensive risk stratification strategy. OARA is an index consisting of nine areas of comorbidities, each weighted based on their expected significance on patient discharge. Meneghini et al^7^ (2017) demonstrated that patients with an OARA score of <59 (low-moderate risk) were twice as likely to be discharged on the same day or the next day, with an 81.6% positive predictive value for early discharge. Furthermore, Ziemba-Davis et al^8^ (2019) demonstrated that patients with OARA score <79 demonstrate a nearly 100% positive predictive value and specificity for same-day discharge TJA. Thus, the data have revealed valuable tools for predicting a patient's ability to undergo outpatient TJA.

Aside from patient-related factors, nonmedical factors also play a role. They include social support (ie, a caretaker to provide help at home); home layout (ie, presence of stairs); and patient motivation, willingness, and understanding of the entire perioperative process. Although there are little data regarding the effect of these factors directly on outpatient TJA, they should be considered for any outpatient program.

Patient selection is an important process in preparing a patient for outpatient TJA. While there is no single score or index to predict success directly, a patient must be evaluated thoroughly and risk-stratified regarding medical and nonmedical risk factors.

## Choice of Anesthetic Techniques

### Neuraxial Anesthesia Versus General Anesthesia

The anesthetic technique used for any TJA surgery carries its own risks and benefits and may influence perioperative outcomes. Commonly used anesthetic options for TJA surgeries include neuraxial anesthesia (spinal anesthesia) and general anesthesia (GA). Spinal anesthesia involves the administration of the local anesthetic directly into the spinal canal and cerebrospinal fluid to induce a blockade of sensation below the desired dermatome level. It is often accompanied by some form of sedation using various intravenous medications to maintain patient comfort during surgery. General anesthesia involves the systemic administration of medication to induce a completely anesthetized and unconscious state, followed by the insertion of an airway device to protect and maintain a patient's respiration during surgery. Many studies have attempted to identify which option is better for TJA, but the results are varied depending on the type of surgery being done, the patient population, and the end point goal of the study.

General anesthesia overall has been associated with a small increase in the rate of complications. Pugely et al^9^ (2013) demonstrated that GA was associated with a small increase in short-term complications associated with TKA (surgical site infection, blood transfusions, and overall complications), worsened by increased comorbidities. Using data from a population-based analysis, Kopp et al^10^ (2017) found evidence for decreased risk of pneumonia, systemic infections, critical care utilization, and inpatient falls when spinal anesthesia was used instead of GA. Furthermore, in a 2019 consensus recommendation from the International Consensus on Anesthesia-Related Outcomes after Surgery group, neuraxial anesthesia received a unanimous recommendation as the preferred method for THA and a majority (42 of 43 votes) recommendation as the preferred method for TKA because of a reduced odds ratio for complications.^11^ These complications include all-cause mortality, pulmonary complications, acute renal failure, stroke, and blood transfusions. Most notably, neuraxial anesthesia was associated with a 24% reduction in thromboembolic events.^11^ Thus, the overall data demonstrate that neuraxial anesthesia is associated with a better overall perioperative outcome for TJA and may be more appropriate in the outpatient setting. In addition, when compared with neuraxial anesthesia, GA has been associated with mild increases in surgical time and postoperative room time in THA.^12^

Despite the lower rates of perioperative complications with neuraxial anesthesia, the REGAIN trial published in 2021 demonstrated nonsuperiority when compared with GA for THA. This randomized control trial showed no difference in primary end points of death or inability to walk independently at 60 days after surgery between the two groups. However, the study groups did show higher rates of perioperative complications from GA, such as pneumonia, acute kidney injury, and critical care admissions.^13^ Thus, with an emphasis on outpatient TJA, neuraxial anesthesia can be considered to minimize perioperative complications and facilitate safe and early discharge.

### Choice of Sedation

Spinal anesthesia alone does not affect a patient's consciousness during surgery; thus, adding sedation or monitored anesthesia care during TJA is crucial for providing a balanced anesthetic and maintaining patient comfort. Common methods of sedation include midazolam bolus, dexmedetomidine infusion, and propofol infusion. Midazolam is a benzodiazepine with rapid and effective anxiolysis when administered intravenously, but elderly patients may be particularly sensitive to its effects.

Choi et al^14^ (2019) demonstrated that a dexmedetomidine infusion could prolong the sensory block level in neuraxial anesthesia for TKA, potentially reducing the need for additional postoperative analgesia medications. In addition, Lee et al^15^ (2020) asserted that a dexmedetomidine infusion attenuated tourniquet-induced metabolic and coagulation effects and decreased the postoperative opioid requirements for patients undergoing TKA under spinal anesthesia. Dexmedetomidine also has a terminal half-life of approximately 2 hours, but prolonged postoperative sedation may be problematic.

Some evidence suggests that using propofol rather than dexmedetomidine or midazolam resulted in a statistically significantly lower incidence of short-term postoperative cognitive dysfunction in adults older than 65 years.^16^ Propofol is an ideal agent for outpatient anesthesia because of its rapid onset of action and rapid recovery. However, it is a profound respiratory depressant, causes a dose-dependent reduction in blood pressure, and can potentiate the effect of other central nervous system depressants. As a result, only properly trained and qualified personnel should administer propofol for sedation.

The choice of sedation that accompanies spinal anesthesia for TJA can have an important effect on perioperative outcomes. Overall, the risks and benefits of each drug must be considered and chosen judiciously for any outpatient program.

## Choice of Analgesic Techniques

An important concern for any outpatient surgical program is achieving adequate analgesia throughout the perioperative period. This is even more true for TJA as patients are encouraged to undergo early ambulation and physical therapy, often within hours after surgery. Thus, optimal perioperative analgesic regimens for outpatient TJA must achieve adequate pain control without causing excessive sedation and/or muscle weakness.

### Multimodal Analgesia

Surgical trauma releases various inflammatory mediators that contribute to nociception, including prostaglandins, histamine, and bradykinin. Furthermore, pain sensitization and potentiation occur by the release of excitatory amino acids (glutamate and aspartate) and from the upregulation of various genes [c-fos, nitric oxide synthase, and cyclooxygenase 2 (COX-2)]. The goal of multimodal analgesia is to use medications that minimize this release and upregulation of pain mediators and potentiators, reducing both nociception and opioid requirements for postoperative pain control.^2^

These medications include anti-inflammatory drugs, such as nonsteroidal anti-inflammatory drugs and acetaminophen, that act to prevent prostaglandin synthesis. Examples include ibuprofen and ketorolac [nonselective cyclooxygenase (COX) inhibitors] and celecoxib and meloxicam (selective COX-2 inhibitors). Considerations must be made when using medications that inhibit COX because of its effects on impairing kidney function and increased risk of bleeding. The combination of administering an NSAID with acetaminophen has demonstrated superior analgesia than with either drug given separately.^17^

Another unique class of medications used for multimodal analgesia includes gabapentinoids. Gabapentin and pregabalin are analog to gamma-aminobutyric acid and prevent the release of excitatory neurotransmitters (voltage-dependent calcium channels), ultimately resulting in decreased sensitization to nociception.^10^ Considerations must be made when using gabapentinoids in the elderly population because they can potentiate sedation, confusion, and dizziness. However, recent studies debate the effectiveness of gabapentinoids in controlling postoperative pain. A 2016 meta-analysis demonstrated improved pain control at rest and decreased overall opioid consumption using a patient-controlled analgesia regimen,^18^ but there are other studies showing that gabapentinoids had no effect on pain control or analgesic requirements.^19^ Despite the variable results among the current studies, perioperative multimodal analgesia regimens often include gabapentinoid administration.

Another important concept of multimodal analgesia is providing analgesia before the onset of nociception (preemptive analgesia). The goal is to prevent the sensitization of pain that results from surgical stimuli, which can help prevent or reduce the incidence of postoperative neuropathic pain.^19^ As a result, typical perioperative pain regimens for outpatient TJA programs often administer oral medications to patients preemptively on the morning of surgery or at least before arriving in the surgical suite.

### Epidural Analgesia

Historically, epidural analgesia was a common option for postoperative pain control after TJA. This was done using a catheter that infused a local anesthetic with or without an opioid into the epidural space, some allowing for patient demand boluses of medication. Epidural analgesia has demonstrated efficacy in controlling postoperative pain and reducing postoperative opioid consumption. However, it is accompanied by a multitude of adverse effects, including urinary retention, hypotension, pruritus, and motor blockade, as well as limiting the use of postoperative anticoagulation for deep vein thrombosis prophylaxis.^10,20^ These adverse effects can potentially affect ambulation or early participation in physical therapy, thus making it less ideal for use in an outpatient TJA program.

### Local Infiltration Analgesia

Local infiltration analgesia is the direct administration of a local anesthetic and other medications (ie, ketorolac) into the surgical site to provide postoperative analgesia. It is thought to provide analgesia while sparing muscle weakness. There have been mixed results regarding the effectiveness of local infiltration versus other modalities for pain control. A review by Li et al^20^ (2019) examined the use of local infiltration for TKA, which seemed to reduce postoperative morphine consumption versus placebo. The authors asserted that it has similar pain control when compared with epidural catheters or femoral nerve blocks (while preserving quadriceps function). Another review by Li et al^21^ (2018) also showed improved postoperative pain control for TKA with local infiltration when compared with epidural catheters. The most recent PROSPECT guidelines for THA do not lend to the notion of superiority over other methods, but local infiltration provides analgesic effects with no notable adverse effects.^22^ There has yet to be an established standard or consensus regarding what composition of medication is best used for local infiltration analgesia.^19^

The well-demonstrated ability to provide postoperative analgesia with minimal adverse effects, especially in avoiding muscle weakness, makes it an excellent option in any established outpatient TJA perioperative analgesic regimen.

### Peripheral Nerve Blocks

Peripheral nerve blocks (PNBs) are regional anesthesia and analgesia techniques targeting individual nerves or fascial planes. They can be conducted using a single injection or continuous infusion of local anesthetic using a catheter. PNBs improve overall pain control, decrease opioid consumption, facilitate earlier participation in physical therapy and rehabilitation, and may promote earlier readiness for discharge.^10,20,22,23^ There are a variety of different PNBs with sufficient data to demonstrate their effectiveness, but there has yet to be a consensus on what individual PNB or a combination of PNBs constitutes the best option for THA or TKA. This could be because of the complexity of the hip and knee sensory innervation and the limitations of targeting individual nerves (versus blanket coverage using epidural anesthesia). Various PNBs are effective for THA and TKA, but all the risks and benefits must be considered.

The lumbar plexus block (LPB) technique involves injecting a local anesthetic into a field within the psoas major muscle, which consistently covers the femoral, lateral femoral cutaneous, and obturator nerves. This block provides adequate analgesia for both TKA and THA, and it has historically been combined with other blocks with favorable results.^11,22^ However, it is a deep compartment block that can be technically challenging, with an increased risk of block failure or bleeding.^23^ Furthermore, LPB often causes undesirable quadricep muscle weakness resulting in delayed ambulation and participation in physical therapy. As a result, LPB has been replaced with other PNBs for TJA.

Fascia iliaca compartment block is a field block conducted by infiltrating the local anesthetic within the iliacus fascia. The spread of the local anesthetic within this plane reliably blocks the femoral and lateral femoral cutaneous nerves. It is associated with decreased pain scores, decreased opioid consumption, and even slightly decreased LOS, carrying only a small risk of muscle weakness after THA. According to the most recent PROSPECT guideline recommendations, it is the PNB of choice for THA.^22^ Although not many studies exist using FIC for TKA, one study did demonstrate some effectiveness in lowering pain scores and reducing opioid consumption.^23^ In summary, although FIC is the recommended PNB for THA, its usage should be carefully considered for outpatient procedures because of the possibility of interference with early ambulation, physical therapy, and discharge.

A femoral nerve block (FNB) involves injecting a local anesthetic around the femoral nerve after it courses underneath the inguinal ligament. It is one of the oldest and most commonly used PNBs for TJA because of its ease of performing and its ability to anesthetize the anteromedial aspect of the thigh and knee.^23^ FNB can be either a single injection or continuous infusion by the placement of a catheter to extend the duration of analgesia.^10^ It was previously accepted as the benchmark for pain relief after TKA and has been shown to contribute to better long-term recovery after TKA, lower rate of opioid consumption, decreased incidence of postoperative nausea/vomiting, and decreased LOS.^20^ Similar results were found for THA.^22^ However, FNB has been associated with notable muscle weakness, decreased range of motion of the knee, increased risk of falls, and reduced participation in active rehabilitation.^10,20,22,23^ Despite excellent postoperative analgesia, FNB has fallen out of favor for use in ambulatory THA and TKA because of these limitations.^24^

Sciatic nerve blocks (SNBs) are done at various sites either through the transgluteal approach (prone position) or through the anterior approach (supine position). Blocking the sciatic nerve at these locations inhibits sensory innervation to the posterior thigh and terminal branches of the sciatic nerve—the common peroneal and tibial nerve. This blocks sensation and motor to the entire lower leg, save for the anteromedial saphenous distribution. Previously, SNB was used as an adjunct to FNB to provide complete analgesia for TKA. Various reviews and meta-analyses have confirmed that SNB with FNB has demonstrated superior analgesia with reduced postoperative opioid consumption and lower overall pain scores at rest.^10,23^ However, they also noted a notable impairment in ambulation within the first day after surgery. Thus, although the addition of SNB can provide improvement in postoperative analgesia, it is likely not an ideal choice to incorporate into an outpatient TKA regimen.

The adductor canal block (ACB) involves the administration of a local anesthetic into the adductor canal deep to the sartorius muscle, an aponeurosis between various muscles in the middle of the thigh. The deposited local anesthetic bathes and blocks the saphenous nerve, the nerve to the vastus medialis, the medial retinacular nerve, and the articular branches of the obturator nerve resulting in analgesia to the anteromedial aspect of the knee.^23,24^ Various meta-analyses have shown that ACB provides similar analgesic properties to FNB with similar postoperative opioid consumption and pain scores after TKA.^19,23,24^ The location of this block is distal to most of the major motor branches of the femoral nerve, preserving quadriceps function (mild risk of vastus medialis weakness), and, therefore, allows for better knee mobility after surgery.^10,20,23,24^ Furthermore, a recent meta-analysis and review by Sun et al^25^ (2020) demonstrated that a continuous ACB with the placement of a perineural catheter provided longer pain relief, improved pain relief with mobilization, shorter LOS, and greater knee mobility after TKA with similar rates of complications to a single-shot ACB. Thus, ACB seems to be the ideal option for postoperative analgesia for outpatient TKA, providing excellent pain control, allowing for early mobility and participation in physical therapy, and facilitating rapid discharge home.

Quadratus lumborum (QLB) and erector spinae plane (ESP) blocks are novel techniques reported to provide postoperative analgesia for TJA. The QLB block is a technique in which a local anesthetic is injected into the thoracolumbar fascia surrounding the QLB muscle, allowing the spread of medication into the paravertebral space. The advantage of the QLB block is that it can provide analgesia to the hip while sparing quadricep muscle functions.^26^ Although it is a relatively new technique with scarce high-quality evidence of its efficacy, there is some evidence in the literature that it can lower postoperative opioid consumption after THA.^22,26^ The ESP block involves depositing the local anesthetic in the plane between the erector spinae muscle and the transverse process of vertebral bodies. The proposed mechanism of action of the ESP block is either anterior diffusion of the anesthetic into the paravertebral space or interfascial spread toward the posterior rami of spinal nerves.^27^ This technique can provide analgesia while sparing muscle function. In a retrospective cohort study by Xu et al^28^ (2020), ESP catheters allowed for greater ambulation distance on postoperative day 1 after THA while maintaining similar opioid requirements compared with FIC catheters. These two new blocks have few large randomized controlled trials or meta-analyses, but there are promising results from small trials and case reports that, with additional high-quality studies, may allow these blocks to be incorporated into an outpatient perioperative pain regimen for THA.

Overall, the choice of analgesia for TJA is vital to the success of an outpatient program. Considering the entire perioperative process, using preemptive and multimodal therapies and PNB for analgesia are vital components of enhanced recovery after surgery (ERAS) protocols.

## Enhanced Recovery After Surgery

Enhanced recovery after surgery (ERAS) is a set of principles that aim to facilitate and guide various medical care teams of perioperative staff, nurses, surgeons, and anesthesiologists to work together with patients to allow for improved patient recovery, decreased LOS, and increased patient satisfaction. This concept was initially implemented in the 1990s for colorectal surgery but has since expanded to other surgical procedures. ERAS protocols have even proven to reduce LOS in orthopaedic surgeries, including TJA.^29,30^ Thus, the implementation of ERAS protocols may be an asset for the establishment of an outpatient TJA program.

ERAS protocols involve the entire perioperative experience, beginning with the patient and the preoperative period. This involves preparing a patient for surgery by optimizing medical comorbidities and modifiable risk factors. Characteristics such as BMI and preoperative mobility status have been demonstrated to directly affect LOS with TJA.^30^ Optimizing these factors through weight loss and preoperative rehabilitation may improve eligibility for the outpatient TJA. In addition to medical optimization, ERAS protocols also address the mental preparedness of patients for surgery. Preoperative education on the entire surgical experience includes details on what to expect regarding pain after surgery, requirements for discharge, rehabilitation, and the healing process. If patients are properly educated and align their expectations with the reality of surgery, they are more likely to be discharged early.^29^ Before discharge home, patients should also have an established support system available to assist in the postoperative period.

Perioperative ERAS protocols include additional optimization of patients on the same day of surgery. This includes optimizing a patient's hydration status and carbohydrate loading while adhering to the strict preoperative fasting guidelines. For example, despite the usual instructions to fast for 8 hours before surgery, patients may ingest clear liquids up to 2 hours beforehand. Patients who were allowed this option had a higher pain threshold as opposed to those who were not.^29^ Carbohydrate loading involves ingesting a carbohydrate-rich drink (that adheres to ASA fasting requirements) before surgery. In nonorthopaedic surgery, this practice allows for earlier return of intestinal function, decreased LOS, and decreased postoperative nausea/vomiting.^29^

Intraoperative ERAS protocols include anesthetic choices that allow for rapid recovery and fewer adverse effects, administration of opioid-sparing pain management drugs, and surgical strategies to minimize blood loss and the need for blood transfusion. The use of a tourniquet for TKA and infusion of antifibrinolytic medications (tranexamic acid) for both THA and TKA have demonstrated success in minimizing blood loss and decreasing LOS.^29,30^

Postoperative protocols also contribute to early discharge. This includes adequate postoperative analgesia by the previously discussed methods, an adequate thromboembolic prophylaxis regimen, and prevention of postoperative nausea/vomiting. Furthermore, patients should aim to have day-of-surgery mobilization and initiation of rehabilitation and physical therapy. If patients do not meet criteria for discharge on the day of surgery, a continued rapid recovery protocol can help decrease LOS.

Implementation of ERAS protocols will improve the patient's perioperative experience and prepare the patient and the entire perioperative team for surgery. As a result, this may facilitate a more successful TJA outpatient program.

## Conclusion

The number of TJA surgeries is expected to increase substantially in the upcoming decades, especially with an aging population presenting with an increased need for TKA and THA. While there continue to be major improvements in both surgical and anesthetic techniques, the concept of outpatient (same-day discharge or 23-hour observation) TJA is relatively new. Proper patient selection, patient optimization and preparation for surgery, perioperative staff coordination, implementation of ERAS protocols, rapid-recovery anesthetic techniques, blood loss mitigation, and multimodal (opioid sparing) analgesia techniques all contribute to faster recovery after surgery, earlier ambulation and participation in physical therapy, decreased LOS, and better patient satisfaction. The evidence in current literature supports the idea that these techniques can assist in successfully setting up and safely performing TJA surgeries in an outpatient setting.
